# Increased Antiangiogenic Effect by Blocking CCL2-dependent Macrophages in a Rodent Glioblastoma Model: Correlation Study with Dynamic Susceptibility Contrast Perfusion MRI

**DOI:** 10.1038/s41598-019-47438-4

**Published:** 2019-07-31

**Authors:** Hye Rim Cho, Nisha Kumari, Hien Thi Vu, Hyeonjin Kim, Chul-Kee Park, Seung Hong Choi

**Affiliations:** 1 Department of Radiology, Seoul National University Hospital, Seoul National University College of Medicine, Seoul, 03080 Republic of Korea; 2 Center for Nanoparticle Research, Institute for Basic Science (IBS), Seoul, 00826 Republic of Korea; 3 School of Chemical and Biological Engineering, Seoul National University, Seoul, 00826 Republic of Korea; 4 Department of Neurosurgery, Seoul National University Hospital, Seoul National University College of Medicine, Seoul, 03080 Republic of Korea

**Keywords:** Tumour angiogenesis, Cancer imaging, Cancer therapy

## Abstract

When glioblastoma multiforme (GBM) is treated with anti-vascular endothelial growth factor (VEGF) agents, it commonly exhibits tumor progression due to the development of resistance, which results in a dismal survival rate. GBM tumors contain a large number of monocytes/macrophages, which have been shown to be resistant to the effects of bevacizumab. It has been reported that tumor-associated macrophages (TAMs) promote resistance to bevacizumab treatment. Therefore, it is important to target TAMs in the GBM microenvironment. TAMs, which depend on chemokine ligand 2 (CCL2) for differentiation and survival, induce the expression of proangiogenic factors such as VEGF. Dynamic susceptibility contrast (DSC)-MR imaging is an advanced technique that provides information on tumor blood volume and can potentially predict the response to several treatments, including anti-angiogenic agents such as bevacizumab, in human GBM. In this study, we used a CCL2 inhibitor, mNOX-E36, to suppress the recruitment of TAMs in a CCL2-expressing rat GBM model and investigated the effect of combination therapy with bevacizumab using DSC-MR imaging. We demonstrated that the inhibition of CCL2 blocked macrophage recruitment and angiogenesis, which resulted in decreased tumor volume and blood volume in CCL2-expressing GBM in a rat model. Our results provide direct evidence that CCL2 expression can increase the resistance to bevacizumab, which can be assessed noninvasively with the DSC-MR imaging technique. This study shows that the suppression of CCL2 can play an important role in increasing the efficacy of anti-angiogenic treatment in GBM by inhibiting the recruitment of CCL2-dependent macrophages.

## Introduction

Glioblastoma multiforme (GBM) is the most common malignant primary brain tumor in adults, representing approximately 65% of all newly diagnosed malignant gliomas^1^. Even after aggressive therapies, including surgery, external-beam radiotherapy, and chemotherapy, GBM patients have an extremely poor outcome, with only 10% of patients surviving 5 years after the initial diagnosis^2,3^. These dismal survival statistics accentuate the immediate need for new approaches to treat GBM.

The antiangiogenic agent bevacizumab (Avastin) is a recombinant, humanized monoclonal antibody that directly targets vascular endothelial growth factor (VEGF) for the treatment of GBM^4^. Although bevacizumab, alone or combined with other therapies, inhibits tumor angiogenesis and growth, GBM develops resistance to bevacizumab after an initial response to bevacizumab treatment. Eventually, GBM becomes progressively invasive with rapid growth^5^. One of the mechanisms of resistance to antiangiogenic therapy, in particular to bevacizumab, is associated with the myeloid cell infiltration of GBM^6,7^. The histological analysis of GBM revealed a heterogeneous cellular composition of neoplastic and nonneoplastic glioma cells, which form a tumor microenvironment comprising brain-resident microglia, infiltrating monocytes/macrophages, endothelial cells, pericytes, neural stem cells, and other immune cell infiltrates^8,9^. Monocytes/macrophages constitute up to 30% of the tumor mass in both human and murine GBM^9,10^. Moreover, increased tumor-associated macrophage (TAM) densities are correlated with shorter overall survival and with the vascular density within the tumor^10,11^. However, the mechanism by which TAMs affect the development of resistance against anti-angiogenic therapies in GBM remains largely obscure.

Chemokine (C-C motif) ligand 2 (CCL2) is one of the main chemoattractant for macrophages and has been implicated in glioma angiogenesis^12,13^. Macrophages that are recruited by CCL2 directly induce proangiogenic factors such as VEGF^12,13^. These recruited macrophages promote vascularization within tumors^13,14^. Together, these observations suggest that CCL2 is involved in tumor angiogenesis via macrophages. The modulation of CCL2-dependent macrophages in GBM has the potential to increase the treatment efficacy of anti-angiogenic agents such as bevacizumab. Thus, the CCL2-targeted Spiegelmer mNOX-E36 could be useful in this context.

Dynamic susceptibility contrast (DSC) perfusion MRI has been widely used as a diagnostic and prognostic tool in the clinical setting. DSC-MRI can be used to determine the relative cerebral blood volume (rCBV) of the tumor, which has been shown to potentially predict the response to therapy in human GBM^15^. A high rCBV seems to indicate that a tumor is viable and is undergoing active neovascularization^15^. Consequently, this parameter has shown utility for characterizing histopathological features, differentiating brain abnormalities^16^, determining the prognosis of glioma patients^17,18^ based on the glioma grade^15,18^, confirming recurrence or progression^17^, predicting malignant transformation, and differentiating recurrent tumors from chemotherapy- or radiation-induced injury^19^. Therefore, DSC perfusion MRI is a suitable *in vivo* modality to assess the effects of CCL2-mediated TAM recruitment on the tumor vasculature in GBM.

Here, we showed that CCL2 expression resulted in enhanced tumor growth with increased resistance to bevacizumab in a rat GBM model. CCL2 inhibition by mNOX-E36 reduced the macrophage recruitment and was used as a combination therapy with bevacizumab. Subsequently, we observed that both the tumor size and blood volume decreased after combination therapy with mNOX-E36 and bevacizumab. Moreover, the positive correlation between the CCL2 expression level and blood volume in GBM patients also suggests that CCL2 is a potential candidate for combination therapy with anti-angiogenic agents during GBM treatment. The purpose of this study was to assess the effects of CCL2 inhibition as a combination therapy with bevacizumab for the treatment of GBM, which was monitored with *in vivo* DSC perfusion MRI.

## Results

### CCL2 inhibition reduces macrophage migration without directly inhibiting angiogenesis *in vitro*

**Figure 1:**
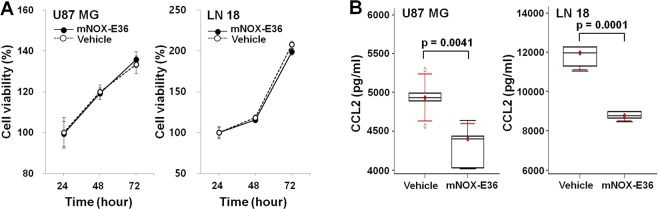
(**A**) The viability of tumor cells was not affected by various incubation times (24 to 72 hours, *n* = 6) with mNOX-E36 (400 µg/mL) in either cell line. (**B**) Intracellular CCL2 expression (*n* = 5 in each group) was decreased in both U87 MG (p = 0.0041) and LN-18 (p = 0.0001) cells after mNOX-E36 treatment. PBS was used as a vehicle treatment (cell lysates were used for the measurement of intracellular levels).

**Figure 2:**
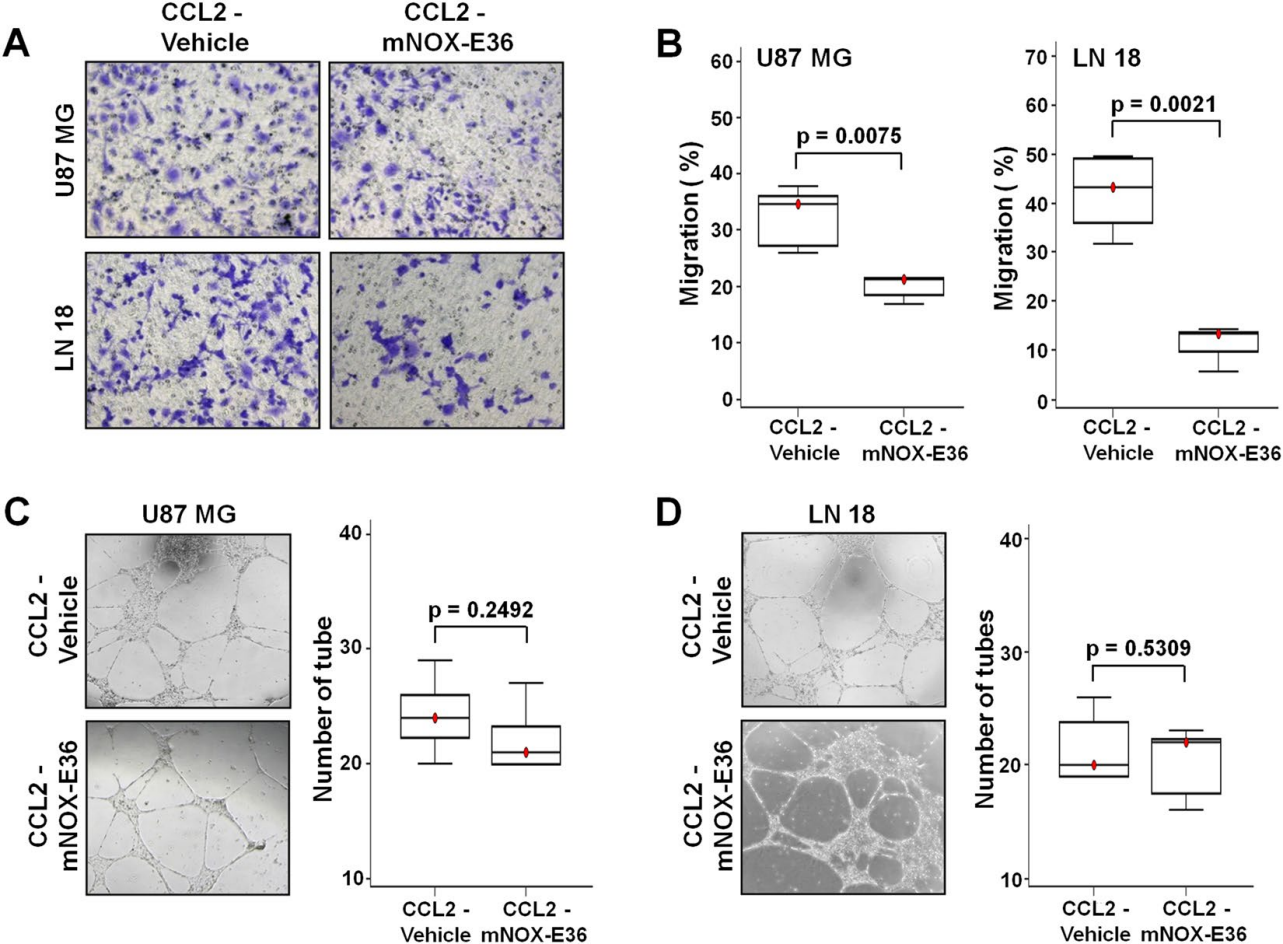
(**A**) The macrophage migration toward mNOX-E36-treated cells was decreased (right) compared with the macrophage migration toward vehicle-treated cells (left) (upper: U87 MG, lower: LN 18). (**B**) Statistical analysis comparing the decrease of macrophage migration (%) toward both the CCL2-expressing U87 MG (p = 0.0075) and LN 18 (p = 0.0021) cells. (**C**,**D**) The angiogenesis assay showed no significant difference between vehicle-treated and mNOX-E36-treated CCL2-expressing tumor cells (U87 MG; p = 0.2492, LN 18; p = 0.5309). The number of tubes that formed during angiogenesis *in vitro* were measured.

To analyze the effect of CCL2 inhibition, we used mNOX-E36 as a CCL2 inhibitor in CCL2-expressing tumor cells. We treated cells with varying concentrations of mNOX-E36 (0–400 µg/mL) for 24 hours, and no difference was observed between the untreated and treated groups at any dose (Supplementary Fig. S1). Further, the tumor cell viability was not changed during 72 hours of mNOX-E36 treatment (Fig. 1A). Decreased CCL2 concentrations were observed at the intracellular level after treatment with mNOX-E36 [U87 MG; Vehicle vs. mNOX-E36 (p = 0.0041), LN 18; Vehicle vs. mNOX-E36 (p = 0.0001)] (Fig. 1B). The migration of macrophages among CCL2-expressing tumor cells decreased after mNOX-E36 (400 µg/mL) treatment compared to that of vehicle treatment [(U87 MG (p = 0.0075) and LN 18 (p = 0.0021)] (Fig. 2A,B). In the angiogenesis assay, the number of tubes generated by *in vitro* angiogenesis was not significantly different between the CCL2-expressing cells that were treated with mNOX-E36 and those that were treated with vehicle (p = 0.2492 in U87 MG and 0.5309 in LN 18, respectively) (Fig. 2C,D). Our findings suggest that CCL2 inhibition by mNOX-E36 significantly decreases the macrophage migration to GBM cells without directly inhibiting angiogenesis.

### Coculture of CCL2-expressing tumor cells with macrophages promotes angiogenesis *in vitro*

**Figure 3:**
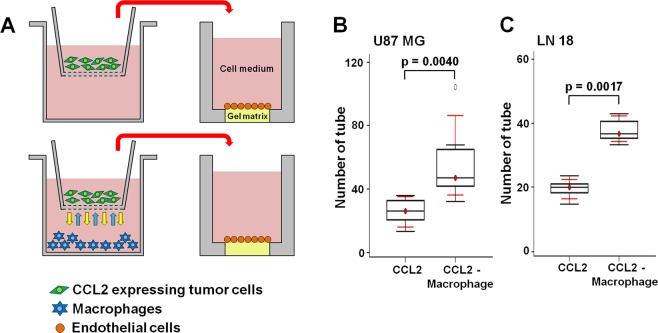
(**A**) The experimental design for the angiogenesis assay. (**B**) The number of tubes that formed is expressed as the angiogenic score, which showed that more angiogenesis occurred in the coculture media from CCL2-expressing tumor cells and macrophages than that from CCL2-expressing tumor cells only for both the (**B**) U87 MG (p = 0.0040) and (**C**) LN 18 (p = 0.0017) cells.

To investigate the association of CCL2 expression with angiogenesis, we used an *in vitro* coculture model (Fig. 3A). In the coculture media of CCL2-expressing tumor cells with macrophages, the number of tubes that formed was significantly higher than that in the culture media from CCL2-expressing tumor cells only [U87 MG; CCL2 vs. CCL2 with macrophages (p = 0.0040), LN 18; CCL2 vs. CCL2 with macrophages (p = 0.0017)] (Fig. 3B,C). This observation suggests that the interactions between the tumor cells and macrophages affects the secretion of CCL2, increasing angiogenesis and resulting in an enhanced number of tubes that form.

### Antiangiogenic resistance increases with CCL2 expression and is suppressed by CCL2 inhibition in a rat GBM model

**Figure 4:**
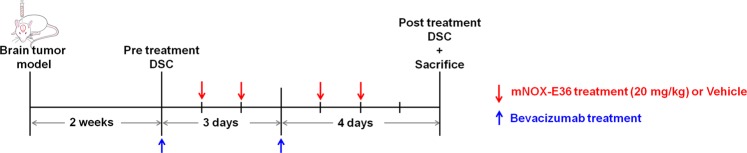
Timeline of the *in vivo* experiments. Abbreviations: DSC; dynamic susceptibility contrast.

**Figure 5:**
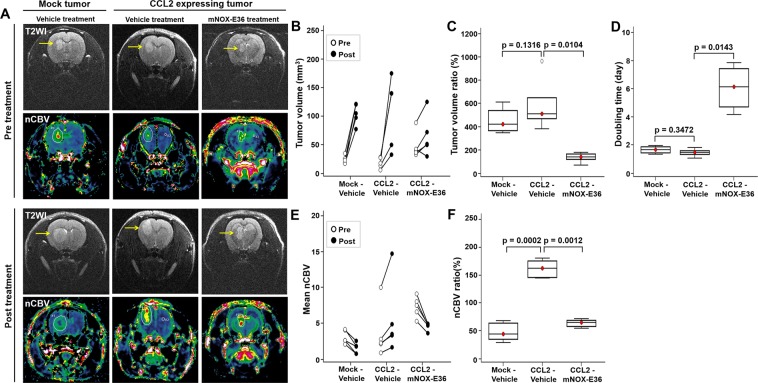
The response of tumors to treatment was evaluated by T2WI and DSC perfusion MRI after combination therapy with mNOX-E36, a CCL2 inhibitor, and bevacizumab in U87 MG tumors. (**A**) T2WI shows the tumor boundaries (yellow arrow), and the nCBV maps obtained with perfusion MRI reveal the vascular changes in the tumors [left column: mock treated with bevacizumab only (Mock-Vehicle); middle column: CCL2-expressing tumor treated with bevacizumab and vehicle (CCL2-Vehicle); and right column: CCL2-expressing tumor treated with bevacizumab and mNOX-E36 (CCL2-mNOX-E36)]. (**B**) The tumor volume increased in all groups after treatment (black circle) compared with the pretreatment volume (white circle). (**C**) The tumor volume ratio (%) of the CCL2-mNOX-E36 group was significantly lower than that of the CCL2-Vehicle group (p = 0.0104). (**D**) The doubling time of the CCL2-mNOX-E36 group was significantly longer than that of the CCL2-Vehicle (p = 0.0143) group. (**E**) The nCBV of the CCL2-Vehicle group increased posttreatment (black circle) compared with the pretreatment nCBV (white circle), but the CCL2-mNOX-E36 group showed decreased nCBV compared to the pretreatment nCBV. (**F**) The nCBV ratio (%) was significantly higher in the CCL2-Vehicle group than in Mock-Vehicle group (p = 0.0002) and in the CCL2-mNOX-E36 group (p = 0.0012). Abbreviations: T2WI, T2 weighted imaging; DSC, dynamic susceptibility contrast; nCBV, normalized cerebral blood volume.

Next, we investigated the anticancer effect of combination therapy with bevacizumab and mNOX-E36 in a rat GBM model, which was monitored with DSC perfusion MRI. For this *in vivo* study, bevacizumab (20 mg/kg, twice/week) with or without mNOX-E36 (20 mg/kg, 4 times/week) was administered intraperitoneally to CCL2-expressing U87 MG tumor-bearing rats after undergoing a pretreatment MRI. A posttreatment MRI was performed after 1 week (Fig. 4). Figure 5A shows representative anatomical T2WI and nCBV maps that were obtained in rats with mock tumors that were treated with bevacizumab and vehicle (left column, Mock-Vehicle) and in rats with CCL2-expressing tumors that were treated with bevacizumab and vehicle (middle column, CCL2-Vehicle) or bevacizumab and mNOX-E36 (right column, CCL2-mNOX-E36). Overall, the tumor volume increased on T2WI (Fig. 5B). However, in terms of the tumor volume ratio (%) and doubling time (DT), the CCL2-mNOX-E36 group had a significantly lower tumor volume ratio and a longer DT than those of the other two groups (Fig. 5C,D).

Furthermore, the change in the nCBV was assessed to monitor the therapeutic effects of the combination therapy. In the Mock-Vehicle group, the posttreatment nCBV decreased compared with the pretreatment value after bevacizumab treatment, but the difference was not significant. However, in agreement with previous findings that showed an increase in tumor volume posttreatment, the nCBV was found to be increased in the CCL2-Vehicle group and to be decreased in the CCL2-mNOX-E36 group after treatment (Fig. 5E) compared to the respective tumor volumes before treatment. In terms of the nCBV ratio, the CCL2-Vehicle group had significantly higher values than those of the Mock-Vehicle group (p = 0.0002), and the CCL2-mNOX-E36 group demonstrated a significant decrease in the nCBV ratio compared with that of the CCL2-Vehicle group (p = 0.0012) (Fig. 5F). However, there were no significant differences in the nCBF, MTT, TTP and leakage ratios among the three groups (Supplementary Fig. S2).

### Infiltration of CCL2-dependent macrophages increases angiogenesis and tumor proliferation *in vivo*, which was suppressed after CCL2 inhibition in a rat GBM model

**Figure 6:**
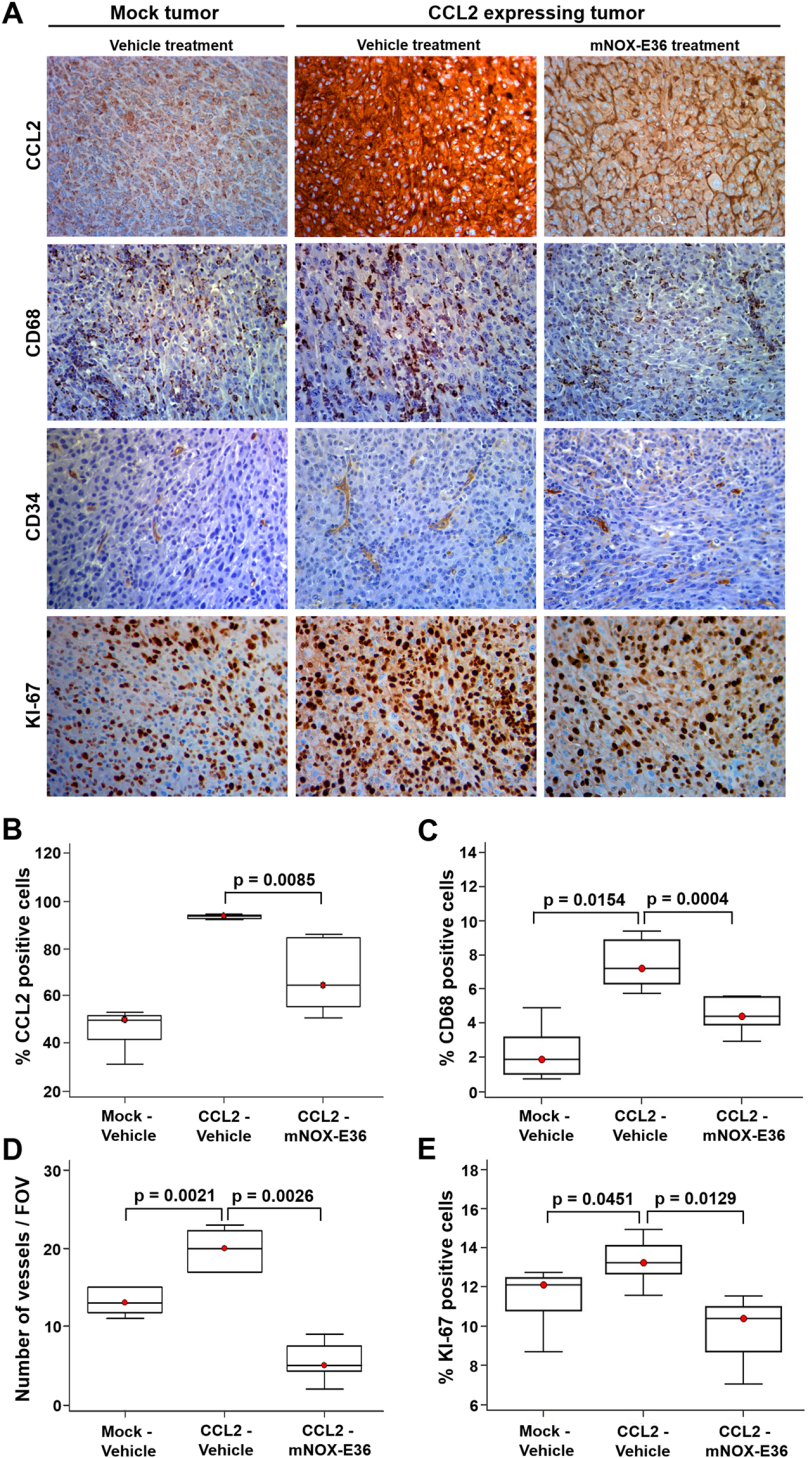
Histological expression analysis of CCL2, CD68 (for macrophages), CD34 (for vascularity), and KI-67 (for cell proliferation) in each tumor (left column: Mock-Vehicle; middle column: CCL2-Vehicle and right column: CCL2-mNOX-E36). (**A**) Expression of all markers was higher in the CCL2-Vehicle group tumors than that in the tumors of the other groups. The first row shows that there was decreased CCL2 expression in the mNOX-E36-treated tumors, which also had a decrease in the recruitment of macrophages (second row), vascularity (third row), and cell proliferation (fourth row) (magnification: ×40). In the quantitative analysis, the CCL2-mNOX-E36 group also showed a further decrease in (**B**) the CCL2 level (p = 0.0085), (**C**) CD68 positivity (p = 0.0004), (**D**) number of vessels (p = 0.0026) and (**E**) KI-67 index (p = 0.0129) in the tissue compared to those of the CCL2-Vehicle group. Abbreviations: FOV, field of view.

Furthermore, to examine the effect of combination therapy with mNOX-E36 and bevacizumab, histological analysis was performed (Fig. 6). We observed that CCL2 expression was significantly elevated in the CCL2-Vehicle group (Fig. 6A,B) compared to that in the Mock-Vehicle group. The number of the recruited macrophages was significantly increased in the CCL2-Vehicle group compared with that of the Mock-Vehicle group (p = 0.0154) and was correlated with the CCL2 expression (Fig. 6C and Supplementary Fig. S3). Moreover, the vascularity and tumor proliferation, as evaluated by CD34 (p = 0.0021) and KI-67 (p = 0.0451) staining, respectively, were higher in the CCL2-Vehicle group than in the Mock-Vehicle group (Fig. 6D,E). However, the tissue CCL2 levels were markedly decreased in the CCL2-mNOX-E36 group compared with those of the CCL2-Vehicle group (p = 0.0085) (Fig. 6A,B). Despite the maintained CCL2 expression, angiogenesis was decreased (p = 0.0026) and macrophage migration was limited (p = 0.0004) in the CCL2-mNOX-E36 group compared to those in the CCL2-Vehicle group. Moreover, the CCL2-mNOX-E36 group had a significantly lower KI-67 index compared with that of the CCL2-Vehicle group (p = 0.0129) (Fig. 6E). The number of necrotic cells, as measured by the release of the enzyme lactate dehydrogenase (LDH) and high-mobility group box 1 (HMGB1) levels, were significantly greater in the CCL2-mNOX-E36 group than in the other groups [LDH: Mock-Vehicle vs. CCL2-mNOX-E36 (p < 0.0001), CCL2-Vehicle vs. CCL2-mNOX-E36 (p < 0.0001); HMGB1: Mock-Vehicle vs. CCL2-mNOX-E36 (p = 0.0007), CCL2-Vehicle vs. CCL2-mNOX-E36 (p = 0.0040)] (Supplementary Fig. S4). Furthermore, C57BL/6 J mice, an immunocompetent mouse model, showed significantly decreased CCL2 expression, F4/80, KI-67, and CD34 expression in CCL2-expressing tumors after mNOX-E36 treatment (Supplementary Fig. S5) compared to those of the CCL2-Vehicle group.

### Long-term survival study in a mouse model with CCL2 expressing GBM

Mice with CCL2 expressing GBM treated with Bev + mNOX-E36 (mean, 32.2 days ± 2.6) had longer survival than that with the Bev + Vehicle treatment (21.5 days ± 3.0; log-rank test, p = 0.0355) (Supplementary Fig. S6).

### Positive correlations among the CCL2 expression, recruitment of macrophages, and nCBV in human GBM

**Figure 7:**
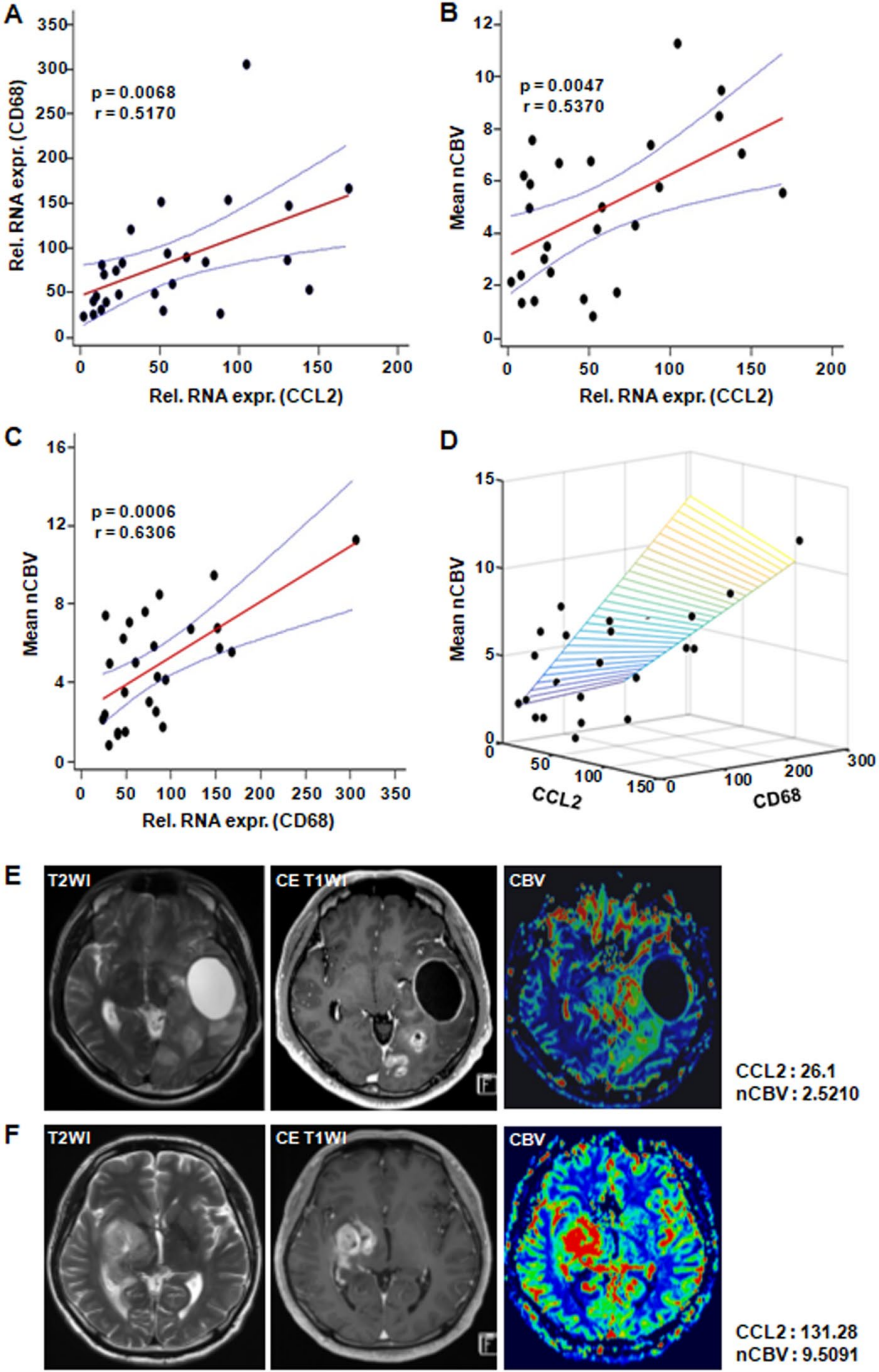
Correlation analysis of the CD68 expression, CCL2 expression and nCBV in patients with GBM. GBM samples (*n* = 26) were analyzed. (**A**) qRT-PCR analysis revealed that the CD68 and CCL2 expression levels were positively correlated. (**B**) A scatter diagram and regression line display the positive correlation between the nCBV and CCL2 (r = 0.5370, p = 0.0047) or (**C**) CD68 expression levels (r = 0.6306, p = 0.0006). (**D**) A 3-dimensional graph of the nCBV, CCL2 and CD68. (**E**,**F**) MR imaging of representative patients who had low and high levels of CCL2 expression and the corresponding correlated nCBV values. Abbreviations: nCBV, normalized cerebral blood volume.

Based on our preclinical *in vivo* results, we performed a correlation study to analyze the association of CCL2-dependent macrophage recruitment with CBV in GBM patients (Table 1 and Fig. 7). CD68 mRNA expression, a marker for macrophages, was significantly correlated with CCL2 expression in GBM patients (r = 0.5170, p = 0.0068). In accordance with our *in vivo* animal data, both CCL2 (r = 0.5370, p = 0.0047) and CD68 mRNA expression (r = 0.6306, p = 0.0006) were significantly correlated with the nCBV as measured by DSC perfusion MRI. In addition, representative cases are also shown in Fig. 7E,F.

**Table 1:** Clinical characteristics of glioblastoma patients.

Characteristics	Total
No. of patients	26
Age (y)*	49.1 ± 13.33
Sex	
Male	19
Female	7
CCL2 level*	55.98 ± 47.96
CD68 level*	84.07 ± 62.25
Tumor size (cm^3^)*	13.01 ± 9.66
mean nCBV*	4.90 ± 2.77

*The data are shown as the mean ± standard deviation.

## Discussion

In most brain tumors, macrophages and microglia are rich sources of stromal factors. Moreover, the fact that as many as 30–50% of the cells in gliomas are microglia or macrophages raises the intriguing possibility that targeting microglia and macrophages might represent an adjuvant therapeutic option for difficult-to-manage cancers^20–23^. The antiangiogenic agent bevacizumab, a recombinant humanized monoclonal antibody directed against VEGF, has been approved by the U.S. FDA for the treatment of GBM^5,24^. Although clinical trials with bevacizumab have shown benefits in regard to response and progression-free survival rates^25,26^, after anti-angiogenic therapy, GBM tumors often exhibit enhanced invasive and infiltrative phenotypes and inevitably relapse^25,27^. Together with these data, several studies have been performed to investigate tumor refractoriness after bevacizumab treatment^28,29^. A recent study by Piao *et al*. showed that the survival of animals treated with bevacizumab in combination with sunitinib was increased compared with that of animals treated with only bevacizumab in an orthotopic mouse cancer model. Additionally, the combined treatment delayed macrophage infiltration until brain tumor progression^30^. Furthermore, Shojaei *et al*. reported that granulocyte colony-stimulating factor (G-CSF) was highly upregulated in refractory tumors of subcutaneously implanted mice^28^. In animals treated with anti-G-CSF, the circulation of tumor-associated myeloid cells was significantly reduced compared to that of untreated animals, demonstrating the important role of G-CSF in tumor refractoriness after bevacizumab treatment^28^. To date, many preclinical studies have indicated that bevacizumab alone does not induce prolonged tumor regression but does generate vessel normalization. Therefore, this treatment might still create a window of treatment opportunity for other agents.

The spiegelmer mNOX-E36, with CCL2-specific binding, is an interesting pharmacological modality because it is highly effective at low nanomolar concentrations, has favorable pharmacokinetic characteristics (based on efficient plasma levels at a once- or twice-weekly dosing schedule), and has no significant immunogenicity *in vivo*^31–35^. Hence, we considered mNOX-E36 to be well suited for the antagonism of CCL2 in the present study, and we used mNOX-E36 to investigate whether resistance to antiangiogenic treatment could be overcome with immunosuppressive therapy that blocked macrophage recruitment in GBM. CCL2 that is produced by the glioma microenvironment is essential for the recruitment of regulatory T cells and myeloid-derived suppressor cells^36^. According to a study by Chang *et al*., elevated levels of CCL2 in clinical specimens of GBM correlated with the reduced overall survival of patients^36^. We believe that mNOX-E36 could be helpful for GBM patients with high CCL2 expression.

In our *in vitro* study, we established CCL2-expressing human GBM cell lines and demonstrated the ability of CCL2-expressing cells to promote angiogenesis via macrophage recruitment. We showed that macrophages did not migrate toward conditioned medium from CCL2-expressing cells after mNOX-E36 treatment. Moreover, our current study suggests that without macrophages, CCL2 expression cannot directly promote angiogenesis. Simultaneously, mNOX-E36, which does not directly affect tumor cell viability, also cannot inhibit angiogenesis. Interestingly, we observed that angiogenesis was increased when both CCL2-expressing cells and macrophages were cocultured to generate the conditioned medium. Based on these results, we hypothesized that the combination of CCL2 secretion of GBM and macrophage infiltration likely plays a critical role in promoting tumor angiogenesis, which encouraged us to perform *in vivo* studies.

In an *in vivo* study, bevacizumab (20 mg/kg, twice/a week) with or without mNOX-E36 (20 mg/kg, 4 times/a week) was administered intraperitoneally to CCL2-expressing U87 MG tumor-bearing rats after performing a pretreatment MRI. The experimental design for the *in vivo* study was based on our previous study, which showed bevacizumab resistance with the same U87 GBM nude rat model. In a previous study, we injected the bevacizumab on the same days as in current study and assessed resistance by DSC perfusion MRI, without CCL2 inhibition treatment. Based on these results, we used bevacizumab before treating with a CCL2 inhibitor in the current study^37^. Furthermore, several studies have shown that bevacizumab does not work effectively alone, as mentioned in relationship to bevacizumab resistance and macrophage recruitment^30,38,39^. One recent study showed that reduced MIF expression was observed in bevacizumab-resistant glioma cells, which in turn was associated with increased M2-like TAM recruitment and the promotion of tumor progression and further resistance to anti-angiogenic treatment^38^. According to Lei Deng *et al*., an SDF-1 inhibitor could reverse the recruitment of macrophages, which in turn potentiated anti-VEGF treatment by bevacizumab and led to survival benefits^39^. In another study, Yuji Piao *et al*. characterized the change in tumor vasculature, cell proliferation and changes in the microenvironment, including the infiltration of different myeloid cell populations, which accounted for the differential outcomes observed with anti-VEGF treatment. The authors suggested that the ability to disrupt the recruitment of bone marrow-derived cells to gliomas is the most important mechanism for obtaining the beneficial effects of anti-angiogenic therapies^30^.

Considering that our study was based on immunotherapy, we used histological analysis without DSC perfusion MRI in an immunocompetent CCL2-expressing C57BL/6J GBM mouse model. According to previous studies, a C57BL/6J GBM mouse model that was generated with GL261 cells closely mimicked GBM phenotypes and was partially immunogenic, as the GL261 cells expressed high levels of MHC1. For this reason, recently, many researchers have preferred to use this model to perform immunotherapy-based research^40–44^. Unfortunately, the bolus injection of a contrast agent is difficult in mice due to their very small veins. However, the expression level of CCL2, proliferation, and vascularity in the C57BL/6J GBM mouse model were significantly decreased after mNOX-E36 treatment compared to those of the controls, and these results were consistent with those of the nude rat model, even though the MR study was not carried out (Supplementary Fig. S5).

Technically, several studies have reported that bioluminescence imaging (BLI) reveals slow tumor growth after anti-CCL2 treatment with docetaxel in mouse prostate cancer. However, the BLI photon counts over time showed no differences between the control and anti-CCL2-treated groups. As the error bars were relatively large, the inclusion of additional numbers of animals may allow for a more definitive conclusion to be drawn^35^. Another study by Rozel *et al*. using diffusion-weighted (DW)-MRI showed differential effects among docetaxel, anti-CCL2 and a combination of docetaxel and anti-CCL2 treatments on PC3 tumor growth in a mouse bone tumor model. The authors reported that the diffusion measurements of the PC3 tumors increased over time based on the water apparent diffusion coefficient (ADC) values in response to both docetaxel and combined docetaxel and anti-CCL2. However, the DW-MRI results did not show that the anti-CCL2 treatment inhibited the infiltration of macrophages at the tumor site^45^. Our study may also be of interest for the development of future imaging strategies for the management of patients who are undergoing GBM treatment. In this study, DSC perfusion MRI was used to monitor the anti-angiogenic effect due to the inhibition of the migration of CCL2-induced macrophages in bevacizumab-resistant GBM. DSC perfusion MRI allows the calculation of physiological parameters such as blood volume, blood-to-tissue transfer constant, and blood-brain-barrier integrity. In addition, this technique can be implemented to measure the size and density of the tumor vessels and offers additional important information^46–49^. We evaluated the changes in vascularity, such as vessel density, rather than vascular permeability after each treatment, which can be evaluated by using DSC- and DCE-MRI, respectively^30,50^. Moreover, a decrease in tumor vascularity is known to be associated with bevacizumab treatment, which can be robustly correlated with pathologic findings. Therefore, DSC perfusion MRI is one of the best tools to analyze the antiangiogenic response related to the CCL2-induced recruitment of macrophages. Furthermore, it is important to establish a method that can allow continuous monitoring of the treatment response and/or recurrence in GBM patients using DSC perfusion MRI. An increased CBV value frequently suggests poor prognosis in GBM patients, which can be affected by several genetic and microenvironmental characteristics. Our animal and human studies showed that the CBV value in GBM is affected by macrophage recruitment, and the response to modulatory therapies can be monitored by DSC perfusion MR imaging.

However, there are several limitations in this study. First, bevacizumab sequesters only human (tumor-derived) VEGF. The previous studies showed that the mouse microenvironment could have secreted mouse VEGF in bevacizumab-treated animals and thus prevented excessive hypoxia, which was found to be associated with re-infiltration of macrophages^30^. Some evidence exists that the elimination of all VEGF from tumors may paradoxically promote tumor growth^51^. We believe that a future study is warranted to determine the level of VEGF that is required for preventing or delaying this unwanted effect as well as to investigate the potential role of rodent VEGF on human cancer cells. Moreover, the concentration of bevacizumab used in this study to induce the bevacizumab resistance was determined according to our previous study^37^. However, the concentration was higher compared to the human dose. Second, we performed a survival analysis study to evaluate the benefit of mNOX-E36 in a CCL2-expressing tumor animal model, thus in future applications of our results, it will be helpful for selected candidates with high CCL2-expressing glioblastomas. Thus, we compared the survival of animals between the Bev + Vehicle and Bev + mNOX-E36 groups. In addition, we minimized the number of enrolled animals to comply with our institutional policy. Third, further clinical investigation is needed to validate our findings. To date, mNOX-E36 has been evaluated in a phase II study in a patient with diabetic nephropathy, and it has not yet been approved for cancer patients^52^. We expect that, in the future, mNOX-E36 will be a relevant method to validate our therapeutic strategy of CCL2 inhibition in GBM patients.

In summary, this study shows that the suppression of CCL2 can play an important role in increasing the anti-angiogenic treatment efficacy in GBM by inhibiting the recruitment of CCL2-dependent macrophages in a rodent model, which was successfully monitored with DSC perfusion MRI. In addition, CCL2 expression is likely related to the aggressive nature of GBM, which may be crucial for selecting tailored treatment for CCL2-expressing GBM patients.

## Materials and Methods

The *in vivo* study was approved by the institutional animal care and use committee of Seoul National University Hospital. Moreover, this retrospective human study was approved by the institutional review board of Seoul National University Hospital, and informed consent was waived. All research was performed in accordance with the relevant guidelines/regulations at our institute. Detailed methods are available in the online supplement.

### Rat CCL2-expressing cell line

To establish a rat CCL2-expressing GBM cell line, rCCL2 (GenBank accession number NM_031530.1) cDNA was PCR amplified and cloned into a GenTarget lentiviral expression vector. The vector contains a GFP-Puromycin fusion dual marker under the control of the RSV promoter. The cloned insert was expressed under an enhanced constitutive CMV promoter (Supplementary Fig. S7). The primers for rCCL2 cDNA cloning were as follows:

rCCL2-F, 5′-AGCCTCCGGACTCTAGAGG-3′,

rCCL2-R, 5′-GCGGCATCAGAGCAGATTG-3′.

To express the rCCL2 transgene in cells, the U87 MG and LN 18 cell lines were transfected with lentivirus and were analyzed by fluorescence microscopy using green filters (Leica, Wetzlar, Germany). rCCL2 expression was confirmed by western blotting, immunocytochemistry, and cytokine assays (Supplementary Figs S8 and S9).

### CCL2 antagonistic Spiegelmer mNOX-E36

The CCL2 binding Spiegelmer mNOX-E36 (5′-GCGACAUUGGUUGGGCAUGAGGC-GAGGCCCUUUGAUGAAUCCGCGGCCA-3′) was modified at the 3′-terminus with 40-kDa polyethylene glycol and was synthesized at NOXXON Pharma AG (Berlin, Germany)^36^. In this study, mNOX-E36 was diluted in PBS for the *in vitro* studies and in 5% glucose for the *in vivo* studies.

### Short-term *in vivo* treatment response study in a rat GBM model

We used 15 male athymic nude rats (6–8 weeks, Harlan) to generate an orthotopic brain tumor model using human GBM cell lines. Rats were anesthetized with a mixture of zolazepam and xylazine and were placed in a stereotaxic device. The U87 MG GBM cell line (3 × 10^6^ cells/3 μl of serum-free RPMI) was inoculated into the right caudate-putamen region. Then, the *in vivo* MRI study was performed as shown in Fig. 1 and as described in a previous study^34^. Two weeks after cell implantation, the pretreatment T2-weighted imaging (T2WI) and DSC perfusion MRI were performed. Then, the GBM rats were divided into three groups (*n* = 5 in each group): a. Mock-Vehicle (mock U87 MG tumor treated with 20 mg/kg bevacizumab and 5% glucose (vehicle)); b. CCL2-Vehicle (CCL2-expressing U87 MG tumor treated with 20 mg/kg bevacizumab and vehicle) and c. CCL2-mNOX-E36 (CCL2-expressing U87 MG tumor treated with 20 mg/kg bevacizumab and 20 mg (based on oligonucleotide weight)/kg body weight mNOX-E36). mNOX-E36 and bevacizumab were injected intraperitoneally (i.p.) four times and two times, respectively, for one week^37,53–56^. The concentrations of bevacizumab^40,57^ and mNOX-E36^58,59^ used in this study were determined according to a previous study. Then, the brains were removed for histological analysis after the posttreatment DSC perfusion MR imaging acquisition.

### Long-term survival study in a mouse GBM model

For long-term survival analysis, we used 12 male nude mice (6–8 weeks) to generate an orthotopic brain tumor model using the CCL2-expressing U87 MG GBM cell line. Mice were anesthetized with a mixture of zolazepam and xylazine and were placed in a stereotaxic device. The U87 MG GBM cell line (10^6^ cells/3 μl of serum-free RPMI) was inoculated into the right caudate-putamen region. Two weeks after cell implantation, a pretreatment MRI (only T2WI) was performed to confirm tumor growth. Then, the mice were divided into two groups (*n* = 6 in each group) according to tumor size, which was equally distributed between the two group: a. Bev + Vehicle (CCL2-expressing U87 MG tumor treated with 20 mg/kg bevacizumab and vehicle) and b. Bev + mNOX-E36 (CCL2-expressing U87 MG tumor treated with 20 mg/kg bevacizumab and 20 mg (based on oligonucleotide weight)/kg body weight mNOX-E36). mNOX-E36 and bevacizumab were injected in the same manner as described above for the duration of the follow-up period. All mice were observed until euthanasia or the survival endpoint of 35 days.

### Acquisition of patient samples

Tissue samples from 26 patients with histologically confirmed WHO grade IV glioblastoma were obtained during surgery. The patients underwent gross total resection followed by concurrent chemoradiotherapy with temozolomide and adjuvant temozolomide for six cycles. All tumor samples used in this study were snap-frozen in liquid nitrogen as soon as possible during the surgery and were stored at − 80 °C. Histological diagnosis was performed using the criteria described in the 2007 WHO Classification of Tumors of the Central Nervous System^60^. Mutations in patient samples were detected via targeted sequencing, as described previously^61^.

### Image postprocessing and data analysis

DSC perfusion MR images were processed by using commercial software (Nordic ICE and NordicNeuroLab, respectively), in which the CE-T1WI or T2WI were used for structural imaging. The rCBV maps were generated using established tracer kinetic models that were applied to the first-pass data^16^. To reduce the recirculation effects, the ΔR2* (1/T2*) curves were fitted to a gamma-variate function, which is an approximation of the first pass response as it would appear in the absence of recirculation or leakage. We did not use an arterial input function in the perfusion analysis. The dynamic curves were mathematically corrected to reduce the contrast-agent leakage effects. Normalization of rCBV maps was automatically performed using the mean value of the blood volume values outside of the tumor, without any intervention of the observers while making the normalized rCBV (nCBV) maps. Then, the normalized cerebral blood flow (nCBF), mean transit time (MTT), time-to-peak (TTP), and leakage maps were generated for the rat DSC perfusion MR images. The nCBV, nCBF, MTT, TTP, and leakage maps are presented as color overlays on structural images.

One investigator (S.H.C., 14 years of experience in neuroradiology) who was blinded to the experimental or clinical data drew ROIs that contained the entire tumor on every continuous section of the coregistered images. Tumor boundaries were defined with reference to the high-signal intensity areas that were thought to represent tumor tissue on the T2WI. Areas of necrosis, hemorrhage, or macrovessels were first identified in the conventional MR imaging sequences and were excluded carefully from the ROIs. After obtaining the total voxel values of the nCBV of each tumor, the total volume and mean nCBV of each tumor were calculated. In the rat model, the tumor doubling time (DT), measured in days, was calculated by using the following equation: DT = t × log2/(logV − logV_0_), where t is the time between the two measurements and V_0_ and V denote the tumor volume at the initial and last MRI examinations, respectively^62^. Additionally, the tumor volume, nCBV, nCBF, MTT and TTP ratios (posttreatment value/pretreatment value × 100) were also calculated. Tumors with negative DTs were excluded from the DT comparison study among the groups.

All data generated or analyzed during this study are included in this published article (and its Supplementary Information files)

## Supplementary information

SI
