# Supplementary material for: Increased Antiangiogenic Effect by Blocking CCL2-dependent Macrophages in a Rodent Glioblastoma Model: Correlation Study with Dynamic Susceptibility Contrast Perfusion MRI
Source: Sci Rep. 2019 Jul 31;9:11085. doi: 10.1038/s41598-019-47438-4 (PMC6668454; doi:10.1038/s41598-019-47438-4)
Supplement: Supplementary file 1 — SI [file 41598_2019_47438_MOESM1_ESM.pdf]

Supplementary Materials for

Increased Antiangiogenic Effect by Blocking CCL2-dependent Macrophages in a Rodent Glioblastoma Model: Correlation Study with Dynamic Susceptibility Contrast Perfusion MRI

Hye Rim Cho[1,2], Nisha Kumari[1], Hien Thi Vu[1], Hyeonjin Kim[1], Chul-Kee Park[4], Seung Hong Choi[1,2,3,*]

[1]Department of Radiology, Seoul National University Hospital, Seoul National University College of Medicine, Seoul, 03080, Republic of Korea. [2]Center for Nanoparticle Research, Institute for Basic Science (IBS), Seoul, 00826, Republic of Korea. [3]School of Chemical and Biological Engineering, Seoul National University, Seoul, 00826, Republic of Korea. [4]Department of Neurosurgery, Seoul National University Hospital, Seoul National University College of Medicine, Seoul, 03080, Republic of Korea.

***Corresponding Author:** Seung Hong Choi, M.D., Ph.D.

Department of Radiology, Seoul National University College of Medicine

Center for Nanoparticle Research, Institute for Basic Science, and School of Chemical and Biological Engineering, Seoul National University

101, Daehak-ro, Jongno-gu, Seoul, 03080, Korea

Tel: 82-2-3668-7832; Fax: 82-2-747-7418

E-mail: verocay@snuh.org

Materials and Methods

Cell culture

The human GBM cell lines U87 MG, LN18 and the mouse GBM cell line GL261 were chosen for this study. Both human GBM cell lines were obtained from American Type Culture Collection (ATCC, Rockville, MD, USA) and maintained in RPMI with 10% FBS at 37 °C. In case of GL261, the cells were purchased from DSMZ (Leibniz Institute DSMZ-German Collection of Microorganism and Cell Cultures) and cultured in 90% DMEM + 10% FBS + 4mM Glutamine. Rat primary bone marrow macrophages and rat primary brain microvascular endothelial cells were purchased from Cell Applications, Inc. (San Diego, CA, USA) and cultured according to the manufacturer's protocols.

Proliferation assay

To assess cell viability in two different human GBM cell lines, U87 MG and LN 18 were seeded on 96-well plates at an initial density of 5×10^4 cells/well treated mNOX-E36 or DMSO as vehicle, and proliferation was measured 24, 48, and 72 hours. To assess the toxicity, various concentration (0 – 400 µg/mL) of mNOX-E36 was treated 24 hours after cell seeding and measured by cell counting kit-8 (CCK-8, Dojindo). Briefly, 10 µl of 5 mg/mL CCK-8 agent was added and incubated for 2 hours. Then, the amount of formazan formed by viable cancer cells was determined by absorbance at 450 nm on a microplate reader. The number of viable cells was expressed as a percentage of control cells. All treatment conditions were performed in sextuplicate and final concentration for *in vitro* experiments in this study was determined by proliferation assay.

Western blot analysis

rCCL2 protein levels were evaluated by western blot analysis. Cells were lysed in ice-

cold lysis buffer [20 mM Tris-HCl (pH 7.5), 150 mM NaCl, 1 mM Na_2EDTA, 1 mM EGTA, 1% Triton, 2.5 mM sodium pyrophosphate, 1 mM β-glycerophosphate, 1 mM Na_3VO_4, 1 μg/ml leupeptin and protease inhibitor cocktail (Sigma)], and the concentration of lysate protein was evaluated using the bicinchoninic acid method (Pierce Biotechnology, Rockford, IL, USA). Approximately 30 μg of protein was loaded in each lane of a polyacrylamide denaturing gel for electrophoresis. After electrophoresis, the protein was transferred to nitrocellulose membranes for blotting. We used a rabbit polyclonal antibody to rat CCL2 (MyBioSource) and a rabbit polyclonal antibody to β-actin (Abcam). Primary antibodies were detected by horseradish peroxidase-conjugated antibodies (Santa Cruz Biotechnology).

Immunocytochemistry

Cells were seeded on glass coverslips. The next day, the cells were fixed in 4% formaldehyde, rinsed three times in PBS and permeabilized in PBS containing 0.1% Triton X-100. After rinsing with PBS, cells were incubated in 3% BSA for 30 minutes at room temperature for blocking. The samples were incubated for 1 hour with the primary antibody (rabbit antibody to rat CCL2, MyBioSource) and for 1 hour at room temperature with the secondary antibody (Alexa Fluor® 594-conjugated goat rabbit-specific antibody, Santa Cruz) after mounting with DAPI. For fluorescence imaging, images were taken using a confocal microscope (Zeiss LSM 510 META).

Cytokine array

Cytokine arrays (Merck Millipore) for rat CCL2 detection were conducted according to the manufacturers' protocols using cell lysate and 48-hour cell-conditioned media that were controlled for protein concentration and media volume. To analyze the effect of mNOX-E36 on CCL2 release in cells, CCL2 expressing cells were grown in media supplemented with

400 μM mNOX-E36 or PBS as vehicle. After 2 weeks, whole-cell lysates and cell conditioned were collected and measured.

Macrophage migration assay

Cell suspensions (2 x 10^5 cells/100 μl) were added to the upper chamber of 8-μm pore Transwell inserts (Corning) for 2 hours at 37 °C to attach to the membrane. Transwells were then moved to 24-well plates containing 600 μl of cell-conditioned media and incubated at 37 °C for 24 hours. Non-migratory cells were removed from the upper surface of the membrane by scraping with cotton swabs. Migrated cells were fixed in 4% paraformaldehyde, stained with 0.2% crystal violet and analyzed at x 20 magnification in five randomly chosen microscope fields per filter.

Matrigel in vitro tube formation assay for angiogenesis

An experimental design for the angiogenesis is shown in Figure 3A. First, 10 μl of growth factor–reduced Matrigel (BD Bioscience) was placed in the wells of μ-slides (Ibidi, Germany) and incubated at 37 °C for 30 minutes to allow polymerization. Then, 50 μl of rat brain microvascular endothelial cells (1 x 10^4 / well) was seeded subconfluently onto each Matrigel-coated well and allowed to adhere for 4 hours at 37 °C. Conditioned medium from co-cultured tumor cells with macrophages or without macrophages were prepared, of which 50 μl was added to the 50 μl cell suspension already in the wells, resulting in a final volume of 100 μl. After an 8-hour incubation at 37 °C, tube formation was assessed. The number of tubes formed was quantitated as previously described. Briefly, a connecting branch between 2 discreet endothelial cells was counted as one “tube.”

MR Imaging protocol in a rat GBM model

MRI studies were performed on a 9.4T MR scanner (Agilent Technologies) fitted with a rapid 72 volume transmit coil and a rapid 72 rat brain receive coil. The rats and mice were anesthetized with 1.5-2% isoflurane/oxygen (v/v), and oxygen saturation and heart rate were monitored. Temperature was monitored and maintained at 37 ± 1.5 °C throughout the experiment. First, unenhanced anatomic T2WI (repetition time (TR) msec/ echo time (TE) msec = 2000/45, 90° flip angle (FA), 31x35 mm field of view (FOV), 256x256 matrix, and 12 contiguous 1-mm-thick sections) were obtained in the coronal planes. Then, T2*-weighted DSC perfusion MR images were acquired by using a gradient-echo pulse sequence with the following parameters: TR/TE = 25/5, 10° FA, 31 x 35 mm FOV, 128 x 96 matrix, three contiguous 2-mm-thick sections, 9.6 sec temporal resolution, and total repetition of 70. After an initial ~30-second baseline acquisition, rapid administration of gadoterate meglumine (Dotarem®, Guerbet) at a concentration of 0.1 mmol per kilogram of body weight in 1/5 dilution with saline was performed via the tail vein catheter by using a syringe pump (Harvard Apparatus) at a rate of 1 ml/10 seconds, followed immediately by a 1-ml saline flush at the same injection rate.

Immunohistochemistry

Immunohistochemistry was performed using formalin-fixed paraffin-embedded tumor blocks. Briefly, 4-μm-thick tissue sections were deparaffinized in xylene and hydrated by immersing in a series of graded ethanol. Antigen retrieval was performed in a microwave by placing the sections in epitope retrieval solution (0.01 M citrate buffer, pH 6.0) for 20 minutes; endogenous peroxidase was inhibited by immersing the sections in 0.3% hydrogen peroxide for 10 minutes. Sections were then incubated with primary rabbit polyclonal antibody to rat CCL2/ MCP-1 (MBS691393) or mouse monoclonal antibody to rat CD68

(UM800047, ORIGENE), goat polyclonal antibody to rat CD34 (AF4117), mouse monoclonal antibody to human KI-67 (UM800033, ORIGENE) in Dako REAL antibody diluent (Dako). To investigate the necrosis, rabbit polyclonal antibody to human LDH (bs-3827R, Bioss) and HMGB1 (OABF00245, Aviva Systems Biology) were used as primary antibodies. Staining for the detection of bound antibody was evaluated by DAB and all imaging were analyzed by Image J. Moreover, the blood vessel numbers which were evaluated by CD34 were expressed based on previous study[1].

The whole protocols for C57BL/6J mouse GBM model study

Mouse CCL2 expressing GBM cell line

To prepare mCCL2 expressing C57BL/6J mouse GBM model, we established mCCL2-expressing GBM cell line using GL261. The mCCL2 (GenBank accession number NM_011333.3) cDNA was PCR amplified and cloned into GenTarget's expression lentiviral vector. The vector contains a GFP-Puromycin fusion dual marker under the RSV promoter. The cloned insert was expressed under an enhanced constitutive CMV promoter. The primers for mCCL2 cDNA cloning were as follows.

mCCL2-F, 5′-ATGCAGGTCCCTGTCATGCT-3′,

mCCL2-R, 5′-CTAGTTCACTGTCACACTGGT-3′

To express mCCL2 transgenes in cells, GL261 was transfected with lentivirus and analyzed by fluorescence microscopy using green filters (Leica, Wetzlar, Germany).

GBM model and histology analysis

6-8 weeks C57BL/6J mice (n = 15) were intracerebrally implanted with CCL2 expressing GL261 cells, respectively, as in the rat model. Then after 2 weeks, each groups (mock tumor + vehicle treatment, n = 5; CCL2 expressing tumor + vehicle treatment, n = 5; CCL2

expressing tumor + mNOX-E36 treatment, *n* = 5) were received drugs or vehicle as described in Figure 4. For histology analysis, mouse CCL2 (TA319701), F4/80 (14-4801-82), KI-67 (TA336566), and CD34 (RAM34) were used as primary antibodies.

The whole protocols for human study

RNA isolation and real-time PCR for human study

Total RNA of each human sample was isolated by using the Qiaquick RNeasy Mini kit (Qiagen) according to the manufacturer's instructions, and the quality of the RNA was verified by an Agilent 2100 bio-analyzer (Agilent Technologies). Reverse transcription was performed with RevertAid H Minus Reverse Transcriptase (Thermo). Briefly, reverse transcription was performed in a volume of 100 μl with 2.0 μg of RNA sample, 15 pmol of oligo deoxythymidine primer, 20 μl of 5x RT Buffer, 20 μl of each 2.5 mM dNTP mix, RNase inhibitor, and reverse transcriptase. The RT conditions were as follows: 10 minutes at 65 °C, 60 minutes at 42 °C, 10 minutes at 25 °C, 10 minutes at 70 °C.

Real-time PCR was performed in a Rotor-Genes Q cycler machine (Qiagen) using Rotor-Genes SYBR Green PCR kit (Qiagen) according to the manufacturer's instructions in a total volume of 20 μl. Cycling conditions for CCL2 and CD68, GAPDH genes were 10 minutes at 95 °C, 40 cycles of 20 seconds at 95 °C, 20 seconds at optimal Tm, 20 seconds at 72 °C. The sequences of primers were as follows: CCL2 5'-agcaagtgtcccaaagaagc-3' and 5'-tggaatcctgaa cccacttc-3', CD68 5'-cccacacaggggtctttg-3' and 5'-gatcaggccgatgatgagag-3', GAPDH 5'-ggc attgctctcaatgacaa-3' and 5'-atgtaggccatgaggtccac-3'. To correlate the threshold (Ct) values from the amplification plots to copy number, a standard curve was generated, and a non-template control was run with every assay. All samples were run in duplicate, and the average value was used.

MR imaging protocol in GBM patients

For human MR study, twenty-six patients underwent conventional MR imaging and DSC perfusion MR imaging using a 3T-scanner (Verio; Siemens Healthcare Sector) with a 32-channel head coil. The conventional MR imaging included T1-weighted imaging (T1WI), such as transverse spin-echo imaging, before and after contrast enhancement or multi-planar reconstructed transverse, coronal imaging with a sagittal three-dimensional magnetization prepared rapid acquisition gradient echo (3D-MPRAGE) sequence before and after contrast enhancement, and transverse T2WI with turbo spin-echo sequences. Contrast-enhanced (CE) T1WI was acquired after the intravenous administration of gadobutrol (Gadovist®, Bayer Schering Pharma) at a concentration of 0.1 mmol per kilogram (mmol/kg) of body weight. The transverse spin-echo T1-weighted imaging was obtained with the following parameters: TR, 558 ms; TE, 9.8 ms; FA, 70°; matrix, 384 x 187; FOV, 175 x 220 mm; section thickness, 5 mm; and number of excitations (NEX), 1. We obtained the 3D-MPRAGE sequences using the following parameters: TR, 1500 ms; TE, 1.9 ms; FA, 9°; matrix, 256 x 232; FOV, 220 x 250; section thickness, 1 mm; and NEX, 1. The parameters of the transverse T2-weighted imaging were as follows: TR, 5160 ms; TE, 91 ms; FA, 124-130°; matrix, 640 x 510-580; FOV, 175-199 x 220; section thickness, 5 mm; and NEX, 3.

The transverse DSC perfusion MR imaging was obtained with single-shot gradient-echo echo-planar sequences during the intravenous administration of gadobutrol at a concentration of 0.1 mmol/kg of body weight at a rate of 4 mL/sec using a power injector (Spectris; Medrad). A 30-ml bolus injection of saline followed at the same injection rate. For each section, 60 images were acquired at intervals equal to the TR. The parameters were as follows: TR, 1500 ms; TE, 30 ms; FA, 90°; matrix, 128 x 128; section thickness, 5-6 mm; intersection gap, 1 mm; FOV, 240 x 240 mm; sections, 15-20; voxel size, 1.875 x 1.875 x 5 mm^3; pixel bandwidth, 1563 Hz; and total acquisition time, 1 minute 30 seconds.

Statistical analyses

All statistical analyses were performed using a commercial software program (MedCalc version 13.1.0.0, MedCalc Software). A p value < 0.05 was considered statistically significant. Kolmogorov-Smirnov's test was used to determine whether the non-categorical variables were normally distributed. Non-parametric data are presented as the median and interquartile range (IQR, range from the 25th to the 75th percentile), and parametric data are shown as the mean ± standard deviation. According to the results of the Kolmogorov-Smirnov's test, a paired or unpaired Student's t-test, Wilcoxon test or Mann-Whitney U-test was performed, as appropriate, to compare the values between two groups. Bonferroni correction was applied for the multiple comparisons of the values (e.g. a p value < 0.017 was considered statistically significant in three groups comparison). Survival data were analyzed by using a Kaplan–Meier survival analysis with a log rank method of statistics in a mouse GBM model. Pearson's correlation analysis was performed to measure the significance of the association between the nCBV parameters and RNA expression level in GBM patients.

References

1. Phys Med. 2015 Sep;31(6):634-41. Effects of microbeam radiation therapy on normal and tumoral blood vessels.

Supplementary Figures

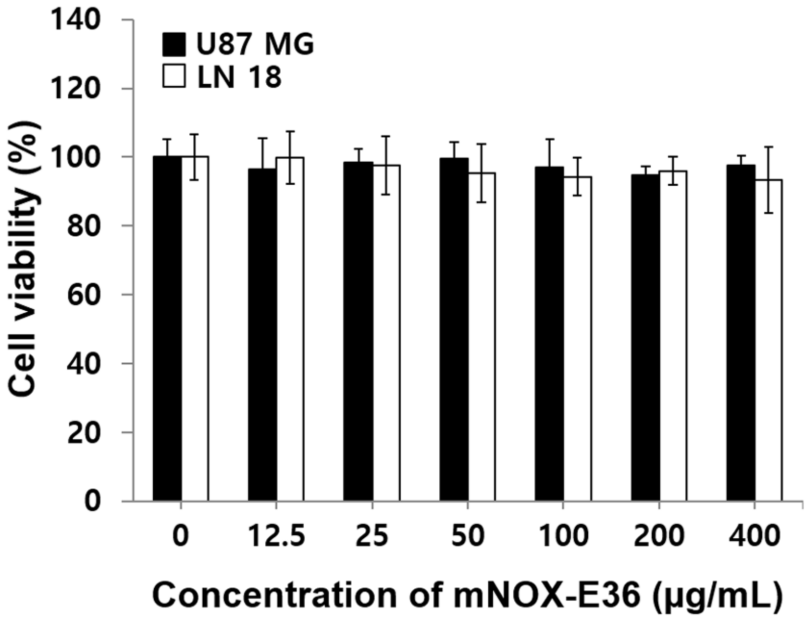

Figure S1. Toxicity test with mNOX-E36 treatment. Cell viability measured with various concentration of mNOX-E36, after 24 hours of treatment. Both tumor cell lines were not significantly different between untreated and 400 µg/mL, which was maximum dose of treatment.

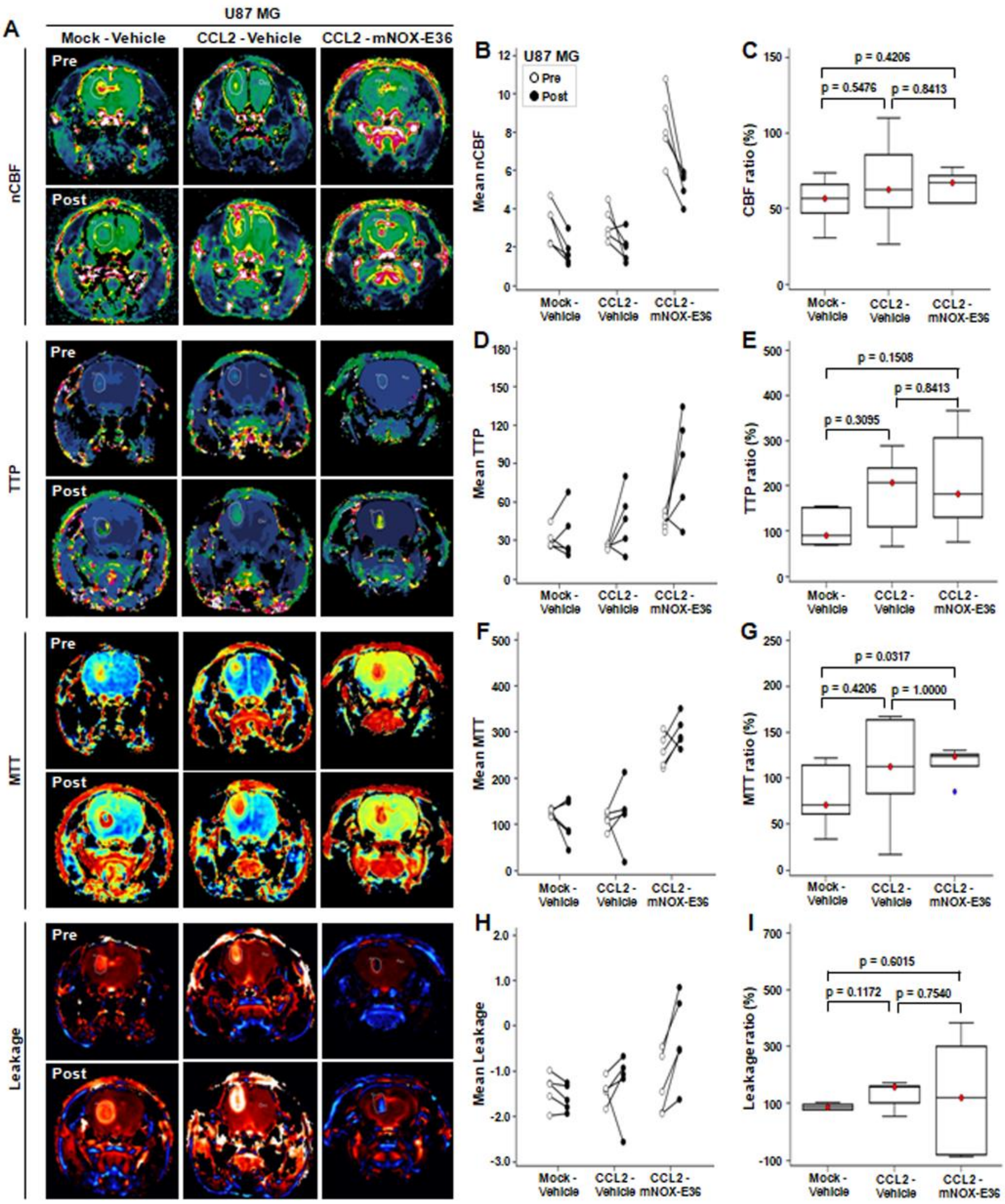

Figure S2. Assessment of nCBF, TTP, MTT and leakage ratios. Changes of nCBF, TTP, MTT and leakage ratios were evaluated by DSC perfusion MRI after combined therapy with CCL2 inhibitor, mNOX-E36, and bevacizumab in U87 MG tumors. (A) nCBF, TTP, MTT and leakage maps obtained by perfusion MRI reveal tumor parameter changes before and after treatment [Left column: mock treated with bevacizumab only (Mock-Vehicle), middle

column: CCL2 expressing tumor treated with bevacizumab and vehicle (CCL2-Vehicle), right column: CCL2 expressing tumor treated with bevacizumab and mNOX-E36 (CCL2-mNOX-E36)]. (B-I) There were no significant differences in nCBF, MTT, TTP and leakage ratios among the three groups. Abbreviations: nCBF; normalized cerebral blood flow, MTT; mean transit time, TTP; time-to-peak

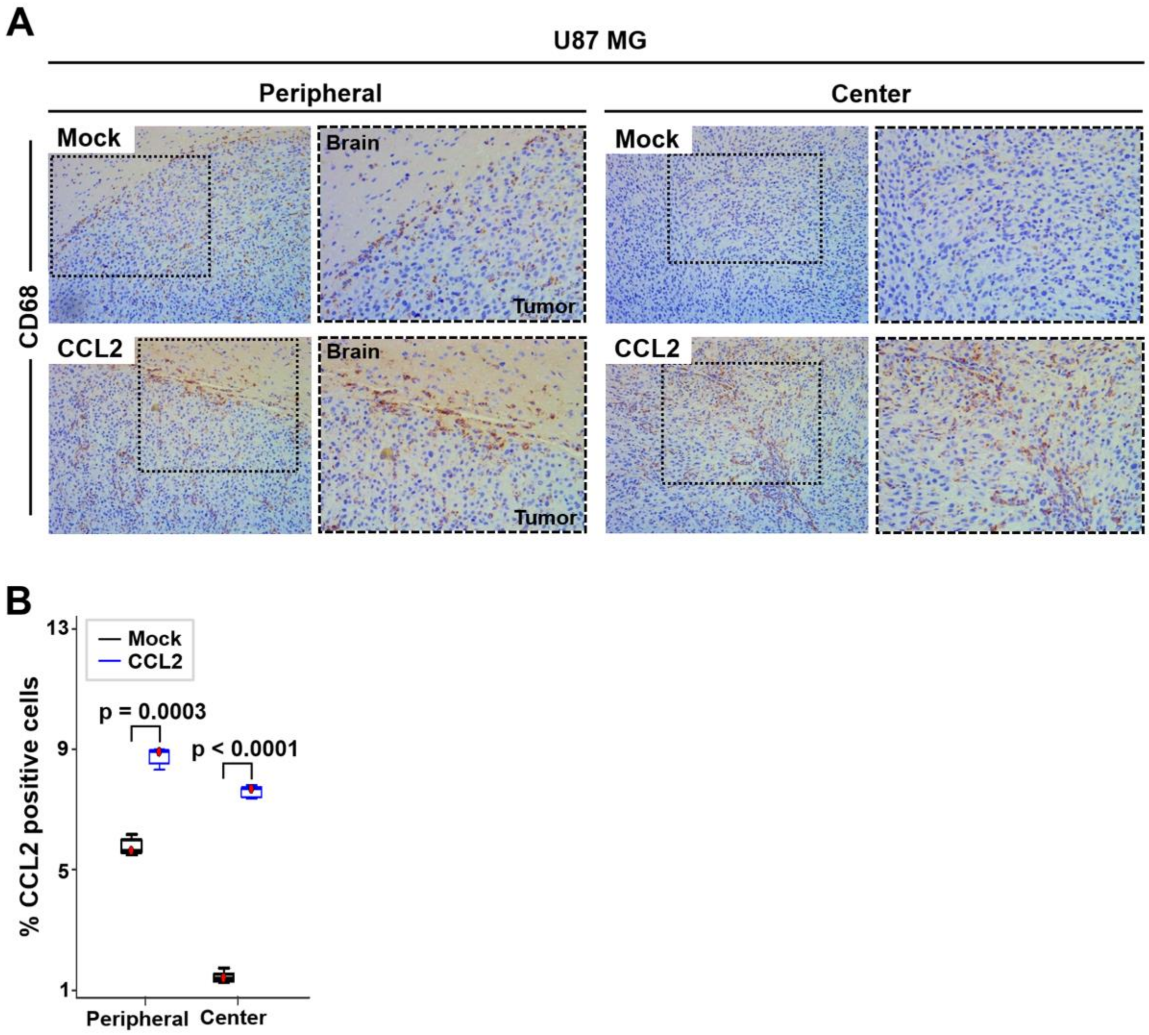

Figure S3. Increased macrophage infiltration in CCL2 expressing GBM. (A) Macrophage infiltration at the peripheral and center in mock and CCL2 expressing GBM bearing rats. The brains were collected from mock (upper) and CCL2 (lower) expressing tumors two weeks after cell implantation and immunostained for macrophage marker CD68. (B) The macrophage infiltration in CCL2 expressing tumors was higher than that in mock tumors in both the periphery ($p = 0.0003$) and center ($p < 0.0001$). Representative figures from each group rats ($n = 6$). Images were captured at ×20 magnification. Higher magnification images are shown in the boxes with black dots (magnification: ×40).

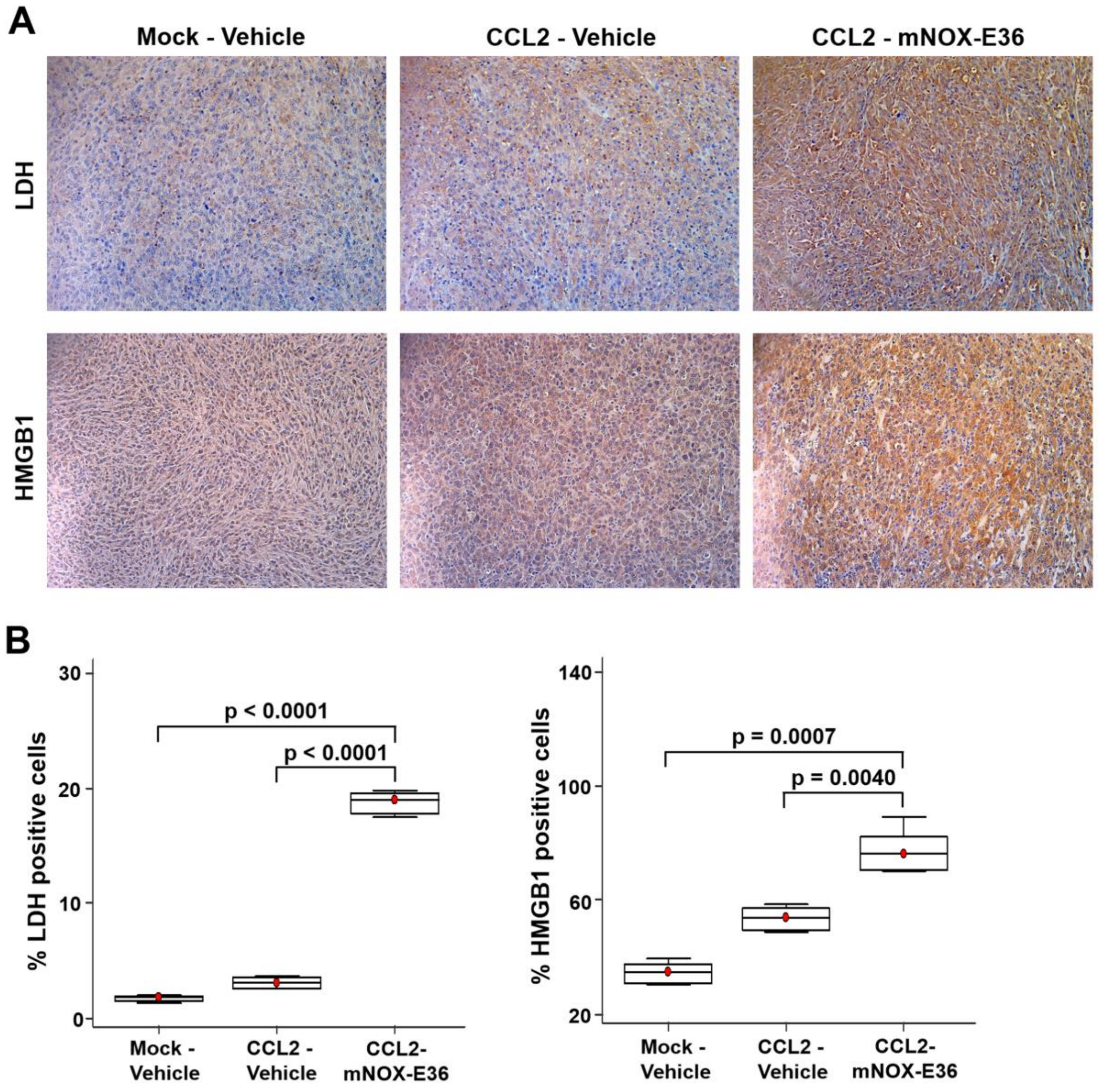

Figure S4. Histology analysis with tumor necrosis factor. (A) The level of LDH and HMGB1 were elevated and (B) the necrotic cells were significantly more prominent in CCL2 expressing tumors treated with bevacizumab + mNOX-E36 than other groups [LDH: Mock-Vehicle vs. CCL2-mNOX-E36 (P < 0.0001), CCL2-Vehicle vs. CCL2-mNOX-E36 (P < 0.0001); HMGB1: Mock-Vehicle vs. CCL2-mNOX-E36 (p = 0.0007), CCL2-Vehicle vs. CCL2-mNOX-E36 (p = 0.0040)]

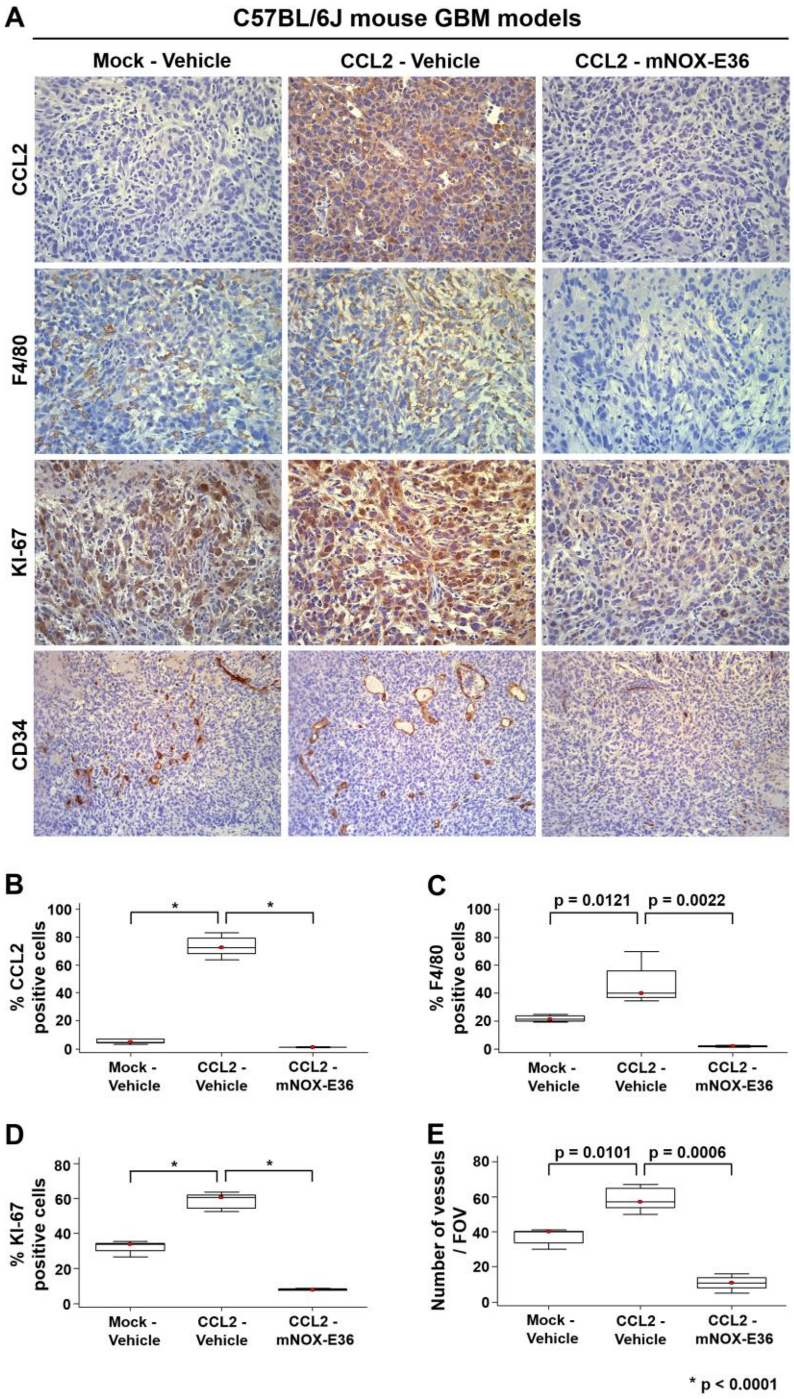

Figure S5. Histology analysis of mCCL2 expressing C57BL/6J mouse GBM model. (A) Immunohistochemistry against CCL2, F4/80, KI-67, CD34 were performed. Magnification for CCL2, F4/80, KI-67: ×40, for CD34: ×20. (B) Positive expression level for CCL2, F4/80, KI-67 and number of vessels by CD34 were quantified. *P < 0.0001.

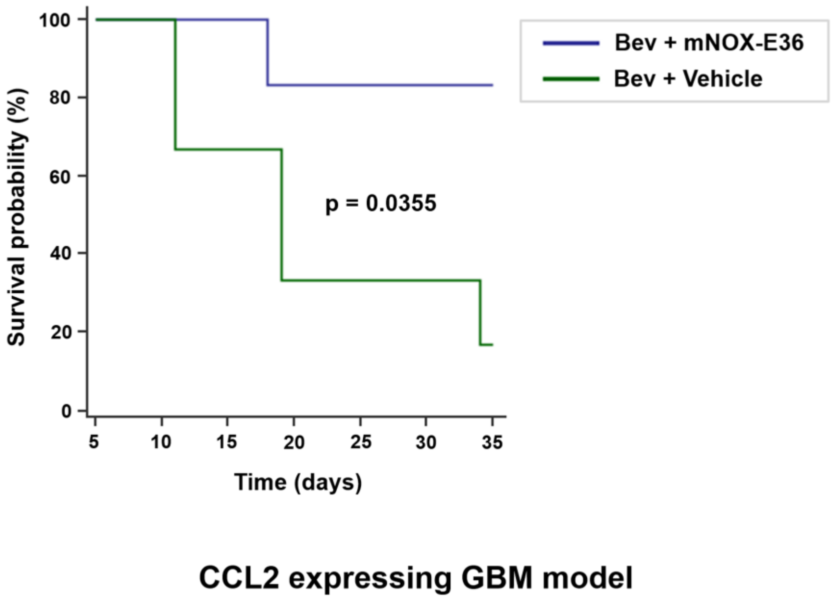

Figure S6. Long-term survival study in a mouse model with CCL2 expressing GBM. The Bev + mNOX-E36 group (mean, 32.2 days ± 2.6) had longer survival than the Bev + Vehicle group (21.5 days ± 3.0; log-rank test, p = 0.0355).

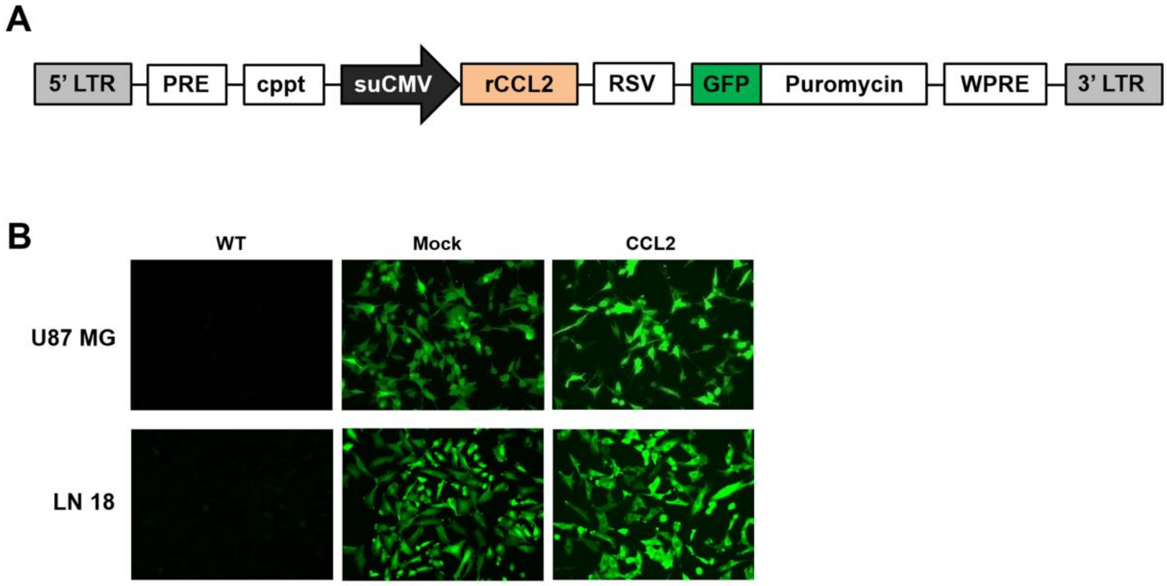

Figure S7. Construction of lentiviral vectors and established cell lines. (A) To generate rat CCL2 expressing lentiviral constructs, CCL2 cDNA was PCR amplified and cloned into GenTarget's lentiviral expression vector (pLenti-suCMV-rCCL2-RSV-GFP-Puro) according to the product protocols. The rCCL2 was expressed under enhanced constitutive CMV promoter, and the GFP-Puromycin fusion was expressed under the RSV promoter in the vector. (B) Infected tumor cells were investigated by GFP expression under the fluorescence microscopy.

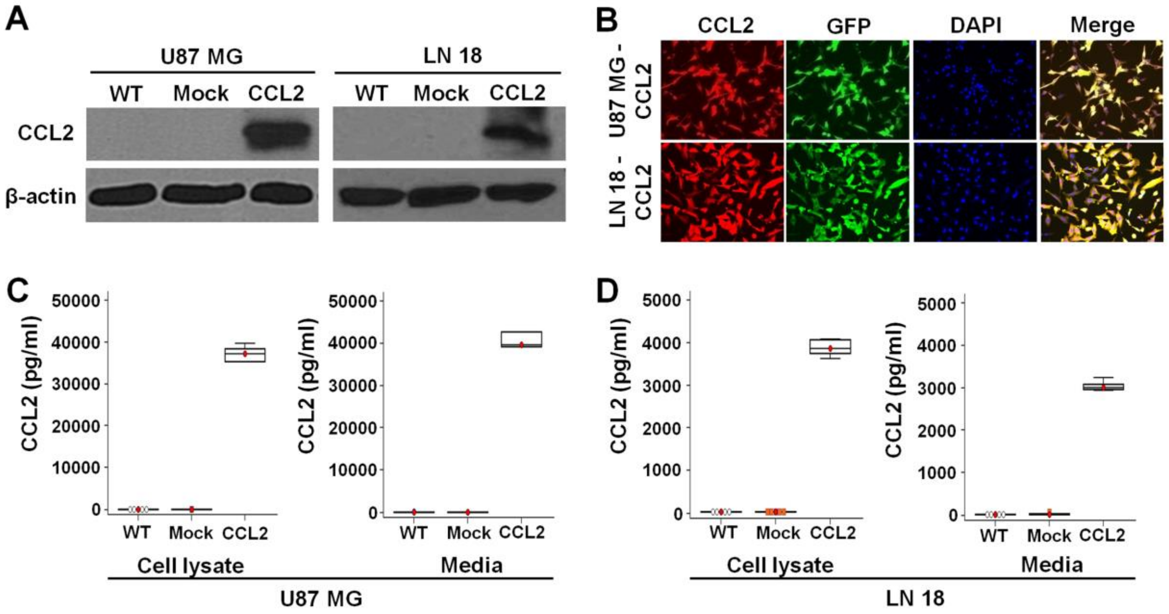

Figure S8. Characterization of established rat CCL2 expressing human glioblastoma cell lines. (A) CCL2 expression was confirmed by western blot (full length western blots are provided in Supplementary Fig. S9) and (B) immunocytochemistry. (C) Intracellular and extracellular CCL2 concentrations in both U87 MG (*n* = 5 in each level) and (D) LN 18 (*n* = 5 in each level) cells were measured by cytokine array and increased in CCL2 expressing cells (cell lysates and media were used to measure intracellular and extracellular levels, respectively).

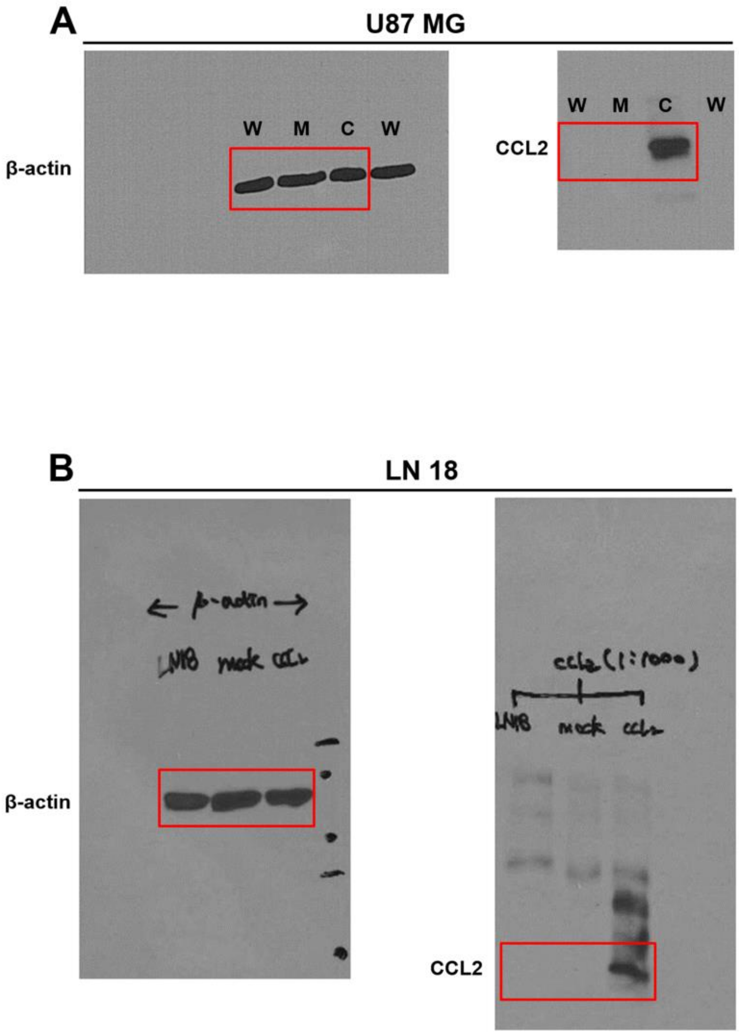

Figure S9. Full length blots for Figure S8. (A) U87 MG (W:WT, M:Mock, C:CCL2) (B) LN 18. The cropped blots for Figure S6 were marked by red square.
